# Fees Versus Salaries

**Published:** 1873-03

**Authors:** 


### Editorial.

***Fees versus Salaries.***

One "Dr. Holland" (whether M.D., D.D., or LL.D., is to us unknown,) is a writer of books seeking fame and fortune, and in the interim of their publication, a writer for newspapers and magazines, with getting the daily bread for the laudable "final cause." In one of his recent temporary flights, he has succeeded in getting noticed by the newspaper press, and hence his magazine article finds larger perusal than it would have secured had it not passed out from the pages where it first saw the light. In this way it has fallen under the Editorial Eye of this JOURNAL, and we are moved to call attention to some few things which intimately concern the medical profession. He assumes that the rewards of professional and literary labor are too largely unequal, the advantage being with the professions. And then, that the lawyer and physician as "a notorious fact" have the advantage of all the other professions. *Ait:*

"There are two forms of income attached to this variety of work, viz., that which arises from salaries, and that which arises from fees. The former is fixed by the community for which the work is done, and the latter by those who do the work. The salaried man enters the market and sells his services at the highest rate which they will command in competition with others. The man of fees combines with his brethren to fix a compensation for his services, which compels the community to take them at his valuation or to do without them."

"The men of fees are the physician and the lawyer. One has to do with the physical diseases of men, and the other with their legal quarrels and their crimes. We do not, in the slightest degree, disparage the usefulness of these two classes of professional men; we simply say that the better the other classes perform their work the less these have to do. They live upon the moral and physical evils of the country; and there is no reason in the nature of their calling for their advantage in pecuniary rewards over the other classes. There is no reason why a general practitioner of medicine, or a specialist in medicine or surgery, shall sit in his office and take in a single fee, for a service that costs him fifteen minutes of time, a sum equal to that which a teacher or a clergyman works all day to win. There is no reason why a physician, called into a house in consultation, should charge for his service a sum that it takes an editor two days of hard work to earn. There is no good reason for the setting of a price upon a surgical operation, performed in half an hour, that the most successful author's copyright cannot pay in a month. It is simple, inexcusable and outrageous extortion. If we go from the physician to the lawyer, we find still higher fees."

etc., etc. But the lawyers may look out for themselves.

That such ineffable *twaddle* should find admission to what claims to be a high toned, first class periodical (*Scribner's Monthly*,) and especially, be paid for, almost surpasses belief. Similar reasoning would put the wages of Dickens, Thackeray, and all other writers of highest repute on the communistic level with the shabbiest penny-a-liner of the daily press. It would put the costly lectures of Beecher and Phillips in the same category of prices with those of the home-made orators of a village lyceum.

If "Dr. Holland" knows anything of a science called Political Economy, he must know that one of its first laws is, that the *Demand* regulates the *Supply*. If the demand is in excess of the supply, prices rise; if less, prices fall.

If the rewards of any particular branch of industry or profession are beyond those of others, then there is a rush into that business, until the equilibrium is restored,

If the medical profession were, on the whole, more lucrative than the others, there would be a rush into its ranks that would speedily bring its emoluments down to par. If physicians find their reward too little, fewer students will seek the doctorate. If all physicians were equally well qualified, and all communities equally well satisfied individually, doctors might become the salaried servants of society. But society pecuniarily would, on the average, gain nothing by the change, for it pays now the same average and no more.

Take any large city, and it will be found that, taken together, the pay received by physicians is not more than that received by any other class, where intelligence is required in business. There are but very few physicians, in town or country, whose income is in excess of the better class of salesmen and bookkeepers in mercantile houses. A very few have large incomes from extensive business and liberal fees—*rari nantes in gurgite vasto.* Of these lucky ones, there are two classes: The mere speculators in disease, the Jim Fisks of the craft; the really meritorious, because educated, sagacious and skillful. These get their large incomes on the simple politico-economical law of supply and demand.

Daniel Webster is said to have replied to a young man who asked him if he thought the legal profession was overcrowded, "There is room enough in the upper stories." Great lawyers, or those with great reputations, ask and receive enormous fees sometimes, yet the pay of lawyers as a class is probably not superior to that of physicians, or any other of the educated or even intelligent classes.

A great artist can demand and get almost any price for his picture or statue, whereas his less fortunately endowed brother in the craft can get little or nothing for the picture or statue he has worked half his lifetime in developing.

It was an Irishman who complained that the railroad charged him as much for riding an hour, as it cost him to ride in a stage-coach all day. Another one who had a diseased molar extricated from its socket deftly by a dentist in a few seconds, demurred to the charge of a dollar, because, on a previous occasion, another dentist charged only half a dollar, although he had been over an hour performing a similar operation, meanwhile dragging him all around the room by the turnkey.

The physician who by a prescription, written in a few minutes, saves a patient's life, or a long siege of sickness, is entitled to little or nothing, compared to the one who with less natural ability, tact, education, and experience, doing his best, even, loses the life, or fails to prevent long and dangerous illness! Such is the necessary inference from the jeremiad of "Dr." Holland.

But space and time forbid our devoting any more thought to this literary chiffonier.

